# Ischemic colitis or melanosis coli: a case report

**DOI:** 10.1186/1749-7922-2-25

**Published:** 2007-09-20

**Authors:** Baber N Chaudhary, Hemant Sharma, Mohammed Nadeem, Mohammed H Niayesh

**Affiliations:** 1 Department of General Surgery, Frenchay Hospital, Bristol, UK; 2 Department of General Surgery, Yeovil District Hospital, Yeovil, UK

## Abstract

**Background:**

Melanosis Coli is described as black or brown discolouration of the mucosa of the colon. Its a benign condition, which arises from anthraquinone laxative abuse and has no symptoms of its own. The main importance of diagnosing Melanosis Coli correctly lies in the fact that if its extensive, there may be difficulty in differentiating it from ischemic colitis.

**Case presentation:**

We present a case of extensive Melanosis Coli involving the whole of large bowel that appeared gangrenous. A sub total colectomy was performed on presumed diagnosis of ischemic bowel.

**Conclusion:**

This report reminds the clinicians that extensive Melanosis Coli may mimic ischemic colitis and thus must be considered as a differential diagnosis.

## Background

Melanosis Coli is a common condition characterized by brown or black pigmentation of colonic mucosa. The condition itself is asymptomatic and the diagnosis is usually made on incidental endoscopic or histological findings. The importance of correctly diagnosing Melanosis Coli lies in the fact that if extensive, it can mimic ischemic mucosa and thus pose a diagnostic dilemma. This clinical situation has been reported earlier in a supposedly necrosed stoma [1,2]. We present a similar but a far more extensive case wherein the entire large bowel mucosa was thought to be ischemic and the patient underwent a subtotal colectomy based on that assumption.

## Case Presentation

**Figure 1:**
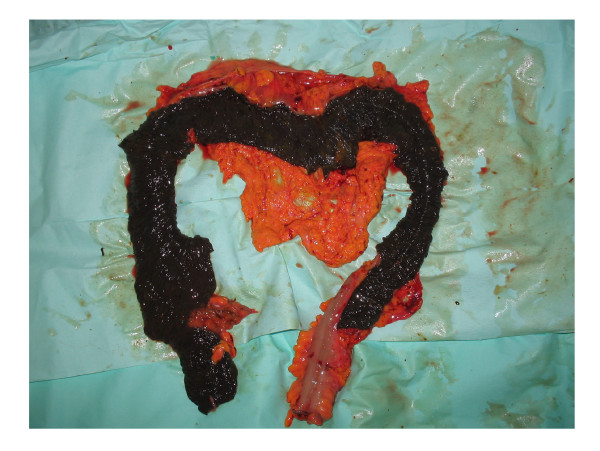
Partially opened specimen showing the very dark mucosa that was mistaken as necrosed. Note the sharp demarcation at ileo-caecal junction.

**Figure 2:**
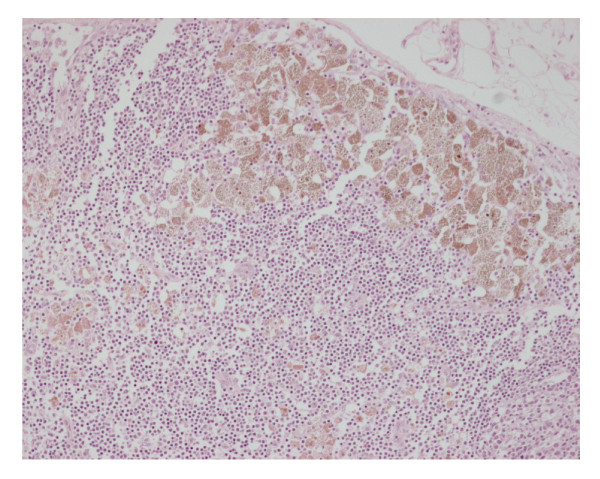
Melanosis Coli in a pericolonic lymph node.

A 63 years old woman presented with a two days history of worsening abdominal pain and distention. She had a past history of deep venous thrombosis, atrial fibrillation [AF] and long standing constipation. Her medications included various herbal laxatives and warfarin. On examination she had quite a distended abdomen with diffuse abdominal tenderness. The blood investigations showed an abnormal white cell count of 14 × 10^3^/dland a CRP of 40. An abdominal CT scan revealed a marked gaseous distention of large bowel with no evidence of stricture or mass lesion. The patient underwent an emergency operation. On laparotomy the large bowel was quite distended and looked akinetic. The mucosa appeared dusky and necrotic from the outside. This abnormal appearance started from caecum and involved the large bowel all the way to the rectosigmoid junction. The pulses, however, were present in the major vessels and the serosa looked moist. There was no obstructing lesion distally and the small bowel was normal. The macroscopic appearance of dusky large bowel mucosa taken together with a history of AF led us to the diagnosis of ischemic colitis. A sub-total colectomy was performed with an end ileostomy. (Figure 1) Patient recovered uneventfully from her operation and was discharged on 6^th ^postoperative day. The histopathology revealed extensive Melanosis Coli with spread to the pericolonic lymph nodes. (Figure 2) There was no evidence of bowel ischemia.

## Conclusion

Melanosis Coli occurs because of the deposition of a brown black pigment called lipofuscin in the lamina propria of the colon [3]. The initial event is mucosal cell death or apoptosis resulting from anthraquinone laxative abuse. These cells are then phagocytosed by macrophages in lamina propria producing lipofuscin, which gives a dark colour to the colonic mucosa.

A variable incidence of Melanosis Coli has been reported in various studies [4,5] with some studies reporting it in well over 50% of the patients. The wide variance probably is because some papers have only looked at macroscopic while others have reported both micro and macroscopic Melanosis Coli. Its incidence is understandably higher in older population [4] and people who suffer from conditions like irritable bowel syndrome and chronic constipation and is rising because of the popularity of the herbal remedies containing anthraquinones [2].

In Melanosis Coli the pigmentation doesn't affect the small bowel [6]. The postulated hypotheses are: lack of anthraquinone receptors in small bowel and conversion of anthraquinones to active metabolites by colonic bacteria. The rectum is involved late.

Melanosis Coli is a benign reversible condition with no malignant potential. The main importance of recognizing this condition correctly is to differentiate it from some of the other more sinister pathologies like ischemic bowel mucosa as highlighted by our case. As per our literature search of English language articles this is the most extensive case of MC ever reported and the only one in which a colectomy was performed on the basis of its appearance.

The histopathology of the specimen not only showed that the there was extensive Melanosis Coli but it had also spread to the pericolonic lymph nodes, again an unusual finding. This is only the sixth case of Melanosis Coli reported in literature where the pigmentation from the bowel had spread to the regional lymph nodes [7].

It's not easy to differentiate Ischemic Colitis from Melanosis Coli when the later is extensive because of various reasons. Firstly, it's very difficult to clinically differentiate Melanosis Coli from Ischemic Colitis when you are doing an operation in emergency circumstances as both conditions can co-exist. Secondly when you encounter this situation in an operation, there aren't any surgical maneuvers or quick tests that would help differentiate between the two. Tests like frozen section of a lymph node or a peroperative colonoscopic biopsy may not be feasible out of hours because of unavailability of a histo-pathologist and time considerations in emergency operations.

The only practical thing which may be of help in such a situation is the awareness that Melanosis Coli can present like this and with that knowledge one can make an informed decision whether there is enough justification to take the colon out or to look for an alternate diagnosis. One of the reasons due to which we went ahead and did the colectomy was the fact that the appearance of the colon was that of ischemic bowel and we didn't know if anything else could also look like that.

This report reminds the clinicians that extensive MC may mimic ischemic colitis and thus must be considered as a differential diagnosis.
